# Efficacy and Tolerability of a Hyaluronic Acid-Based Extracellular Matrix for Labia Majora Rejuvenation and Augmentation: A Pilot Study

**DOI:** 10.7759/cureus.58970

**Published:** 2024-04-25

**Authors:** Azin Ayatollahi, Aniseh Samadi, Behrooz Barikbin, Mohammad Saeedi, Leila Saeedi, Shayan Zamani, Mahsa Fattahi, Alireza Firooz

**Affiliations:** 1 Dermatology, Center for Research and Training in Skin Diseases and Leprosy, Tehran University of Medical Sciences, Tehran, IRN; 2 Dermatology, Laser Application in Medical Sciences Research Center, Shahid Beheshti University of Medical Sciences, Tehran, IRN; 3 Medical Microbiology, Immunology, Asthma and Allergy Research Institute, Tehran University of Medical Sciences, Tehran, IRN

**Keywords:** hyaluronic acid filler, extra cellular matrix, labia major, female genital cosmetic surgery, noninvasive vaginal rejuvenation

## Abstract

A new injectable solution containing low-molecular-weight hyaluronic acid (HA) and a specific amino acid mixture was formulated with proper aesthetic performance for the main signs of facial skin photoaging. The present study aimed to investigate its new application for rejuvenating and augmenting labia majora using clinical and biometric assessments. Three sessions of intradermal injections were performed using 3 ml of test extracellular matrix (ECM) for 10 eligible post-menopause female subjects (age 53.6 ± 7.93 years). The effectiveness of the intervention was assessed by an independent physician using before-and-after pictures based on the physician’s global assessment score. Objective biophysical skin assessments, including skin hydration, skin erythema, and melanin index, as well as elasticity parameters including firmness (R0), gross elasticity (R2), and net elasticity (R5), were also performed before the first injection and then on the 2nd and 12th weeks after the last session. Patients’ satisfaction and all reported or observed adverse events were documented. At week 12, all the subjects reported an aesthetic improvement of 25% or more in rejuvenation and sagging of the labia major area. A statistically significant improvement was also detected in R0 and R5 at week 12 (p-values 0.005 and 0.022, respectively). Patient satisfaction surveys revealed a median score of 8 at both follow-up visits. The results showed a new indication of the tested HA ECM for providing a beneficial, durable, rejuvenating effect on the labia majora with a good safety profile.

## Introduction

The external female genitalia, also known as the vulva, is an organ in women with both reproductive and urinary functions. The labia majora are a pair of cutaneous structures that form a fold that covers the other underlying vulvar structures. Labia majors have an important role in sexual behavior; blood engorgement in this compartment creates a bulging, edematous appearance during female arousal that serves the purpose of sexual attraction to the opposite sex [1]. The morphology and physiology of both the vulva and vagina undergo characteristic age-related changes over a lifetime. The most noticeable changes are due to certain stages of life due to hormonal changes such as puberty, pregnancy, and menopause; these changes can also happen transiently in the case of the monthly menstrual cycle [2].

The change in the appearance of the external genitalia affects sexual self-esteem, and therefore more women tend to seek rejuvenation treatments for the area. There are different methods for rejuvenation of labia majora, including hyaluronic acid (HA) filler injection, lipofilling (autologous fat transplant), surgical methods, platelet-rich plasma injection, and radiofrequency inductions [3-5].

Hyaluronic acid filler injection is a well-known rejuvenation technique, especially for the labia majora aesthetic. It is easy, minimally invasive, repeatable, and reversible with almost no complications, and it is an outpatient choice for women seeking a non-surgical approach to genital rejuvenation [6]. Results have been satisfactory for most patients while having trivial adverse events; in the case of labia majora, augmentation has shown a significant improvement based on the Global Aesthetic Improvement Scale (GAIS) [7,8]. Moreover, the hyaluronic acid injection in the labia site has been associated with the improvement of certain symptoms, such as itching and atrophy-associated friction [9]. 

Sunekos® is a tissue reconstructive product containing low-molecular-weight HA and a selection of amino acids (HY6AA Formula: Glycine, L-proline, L-lysine, L-leucine, L-valine, and L-alanine). It functions by inducing tissue water containment by HA replacement and also extracellular matrix (ECM) bioregeneration through stimulating collagen type IV production via the amino acid components’ effects. Sparavigna et al. studies demonstrated its beneficial effects for rejuvenation and hydration in the head and neck area; besides, there has been supporting evidence for its improving effect on skin elasticity [10]. The present study aimed to investigate a new application of Sunekos® for rejuvenating and lightening labia majora using clinical and biometric assessments.

## Materials and methods

This single-group, before-and-after pilot study was performed on 10 female patients desiring labia majora rejuvenation. Ten healthy post-menopause female subjects were included in this study. All patients were affected by and complained about hypotrophy and hyperpigmentation of the labia majora. Previous rejuvenating or lightening treatment of the area within the last six months, active local infections (bacterial, mycosis, and herpes simplex), treatment with anticoagulants, history of surgery at the site of injection, primary cancer of the external genitalia or metastasis of other cancers to the area, previous regional radiotherapy, and allergic reactions to HA were the exclusion criteria.

Intervention

Three sessions of intradermal injections with one-week intervals were performed using 3 ml of Sunekos®, a medical device (class III) (manufactured and distributed by Professional Dietetics SpA), composed of bottles containing 100 mg of sterile and non-pyrogenic lyophilized glycine, L-proline, L-leucine, L-lysine HCI, L-valine, L-alanine, and sterile vials containing sodium hyaluronate (30 mg in 3 ml of distilled water). No other aesthetic procedure was done along with the intradermal injection of HA. The epilation method used for all participants was waxing; none of the subjects had performed laser hair removal ever before taking part in this study.

Injection technique

For each labia, 1.5 ml of the material was injected intradermally with a 30-gauge mesotherapy needle along the full length of the labium majora. Emla cream 5% was applied to the area with plastic occlusion for 30 minutes before the intervention.

Assessments

The effectiveness of the intervention was assessed by an independent physician using before-and-after pictures based on the physician’s global assessment score.

Objective biophysical skin assessments were performed on three different occasions: once before the first injection, the 2nd week, and the 12th week after the last session. These measurements included skin hydration, skin erythema, and melanin index, as well as elasticity parameters including firmness (R0), gross elasticity (R2), and net elasticity (R5), on the right labia major, by the Corneometer, Mexameter, and Cutometer of the MPA 580 device (Multi Probe Adapter System® manufactured by Courage + Khazaka Electronic GmbH), respectively. 

Before each measurement, the participants rested for 30 minutes in a room with climate control, a temperature of 22±2 °C, and a relative humidity of 30-40%.

All adverse events, either reported by the subjects or observed by the investigators, were documented in each of the treatment and follow-up visits. The main assessed safety parameters were erythema, itching, skin rash, edema, bruising, hypertrophy, pigmentary changes, and paresthesia at the injection site. Their severity was also assessed according to a 3-point scale (defined as 1: mild, 2: moderate, and 3: severe).

Patient satisfaction

Each subject was asked to rate her level of satisfaction based on the visual analog scale (0 to 10).

Statistical analysis

The efficacy and safety of the treatment were analyzed based on the intention-to-treat (ITT) population. For statistical analysis, we performed descriptive statistics (median, range, and percentages). For efficacy assessment, statistical differences were tested between the median score of objective and subjective parameters before intervention and 2nd and 12th weeks after the last injection, using the Wilcoxon Rank Test. The significance level was set at p < 0.05.

## Results

Ten eligible post-menopause female subjects were recruited. The median age was 53.6 ± 7.93 years (range 45-71). No exclusions or loss of follow-up occurred during the study.

Clinical efficacy evaluation

**Figure 1:**
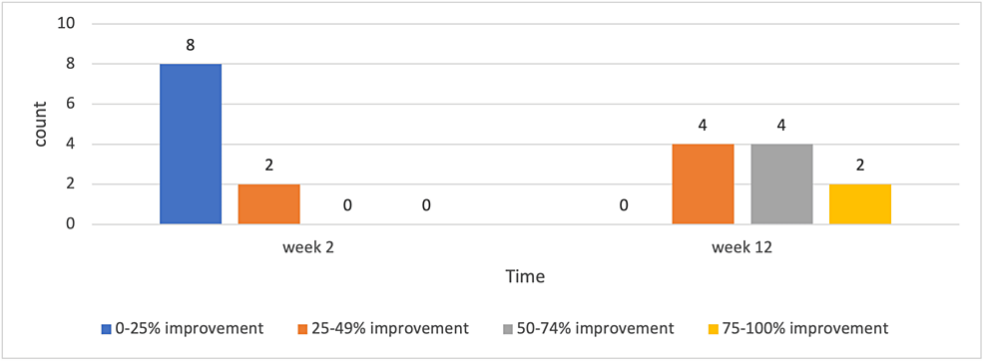
Independent physician aesthetic evaluation of the treated area based on GAIS. GAIS: Global Aesthetic Improvement Scale.

According to the independent physician evaluation based on the physician’s global assessment score, clinical assessments showed improvement in all subjects at both the 2nd and 12th weeks after the injections. Two weeks after the final injection, only 2/10 reported more than 25% improvement. At 12 weeks after the last injection, all the subjects reported an aesthetic improvement of 25% or more in hyperpigmentation and sagging of the labia majora area (Figure 1).

Skin elasticity

At week 12, we detected a statistically significant improvement in R0 and R5 (p-values 0.005 and 0.022, respectively). In the case of R0, the improvement was also reported as significant at week 2 (p-value = 0.005). R2 did not show any significant improvement after the intervention (Table 1).

**Table 1:** Skin biophysical parameters before intervention, at 2nd week post-injection, and at 12th week post-injection R0, R2, and R5 indicate skin firmness, gross elasticity, and net elasticity, respectively. Skin biophysical parameters are in the median (IQR) format. P-value 1: comparison between baseline and 2 weeks after intervention (Wilcoxon rank test). P-Value 2: comparison between baseline and 12 weeks after intervention (Wilcoxon rank test).

Variable	Baseline	2 weeks after intervention	12 weeks after intervention	P-value^1^	P-value^2^
R0 (mm)	0.45 (0.28–0.60)	0.26 (0.09–0.40)	0.30 (0.19–0.41)	0.005	0.005
R2 (%)	0.82 (0.47–0.92)	0.79 (0.05–0.89)	0.80 (0.50–0.91)	0.139	0.721
R5 (%)	0.28 (0.18–0.50)	0.22 (0.02–0.36)	0.30 (0.00–0.69)	0.799	0.022
Skin hydration (arbitrary unit)	36.25 (21.43–69.03)	34.80 (18.03–65.67)	32.71 (19.33–49.60)	0.721	0.241
Skin melanin index (arbitrary unit)	254.50 (152.67–427.33)	261 (114.33–330.33)	280 (138.67–340.67)	0.575	0.646
Skin erythema index (arbitrary unit)	243 (144–360)	243.5 (92.67–457)	228.83 (158–446.67)	0.838	0.445

Skin hydration and pigmentation index

No significant difference was observed between the skin hydration and melanin and erythema index on weeks 2 and 12 (Table 1).

Patient satisfaction

**Figure 2:**
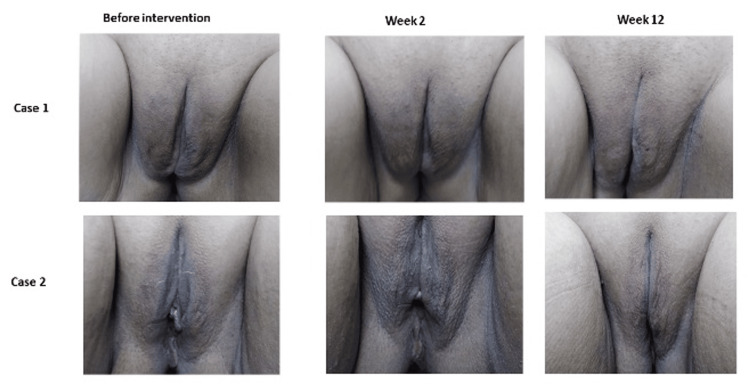
Rejuvenation and lightening effect of the treatment at weeks 2 and 12 on two participants

The median satisfaction scores with the treatment were 8/10 (range 2-10) and 8 (range 3-10), out of 10 on weeks 2 and 12, respectively, which means "appropriate satisfaction" with the treatment process (Figure 2).

Adverse effects

Twenty-seven adverse effect reports were received during the study, which comprised mild erythema, swelling, and burning at the injection area. All the reported adverse effects were totally resolved within 48 hours after the injection. There were no reports of life-threatening events.

## Discussion

This study aimed to evaluate the efficacy and safety of the ECM bio-regenerative agent, Sunekos®, as a novel therapy for labia majora rejuvenation. All subjects showed 25% or more improvement by the 12th week after the procedure. Patient satisfaction surveys revealed a median score of 8 at both follow-up visits.

The literature suggests several methods of labia majora augmentation and rejuvenation [11]. However, efficacy and satisfaction assessment are variable and mostly based on subjective tools.

In accordance with our findings, Fasola and Gazzola described the result of hyaluronic acid injection for labia majora augmentation. A significant improvement (P < 0.0001) in the GAIS score was observed, both in the scores provided by the patients and by the doctor [8]. Zerbinati et al. also reported a significant clinical improvement in labia majora augmentation in the score provided by both patients and doctors after treatment with a similar crosslinked HA dermal filler containing 28 mg/ml PEG [12].

As a confirming method, we used instrumental biometry assessment to evaluate the changes in skin biophysical parameters after treatment. A significant improvement was detected in the skin firmness (R0) and net elasticity (R5) 12 weeks after the last injection session, which objectively supported the subjective results. R0 demonstrates skin firmness, and the higher the R0 values, the more susceptible the skin is to deformity. R5 focuses on measuring the solid components of the skin [13].

Improvement of skin elasticity is primarily interpreted by increasing the skin water content [14]. Moreover, hyaluronic acid, as a major active ingredient in the product, can promote collagenases and elastogenesis at the injection sites. HA interferes with keratinocyte proliferation and migration, facilitates the rearrangement of dermal collagen fibers, and leads to advanced elasticity properties [15].

A comparable objective finding was observed in a study of Sunekos® for facial rejuvenation [10]. Skin hydration did not show significant improvement in our study. It could be explained by the measurement method of the corneometer, which mostly evaluates the water content of stratum corneum, while intradermal injection of the test product apparently affects the deeper layers.

Amino acids are another important ingredient in the test product. In vitro studies have shown that an optimal proportion of amino acids, such as L-alanine and L-valine, can stimulate fibroblasts to increase the production of collagen and elastin in the ECM [16]. Murakami et al. also suggest that combinations of branched-chain amino acids and glutamine or proline are important for restoring dermal collagen protein synthesis impaired by UV irradiation [17].

The findings of this pilot study suggest that Sunekos® is effective for the purpose of labia majora revitalization by improving skin firmness, elasticity, and visual appearance, which can result in satisfactory clinical results both objectively and subjectively. This was the first study to evaluate the efficacy of an ECM bio-regenerative agent for external genitalia rejuvenation; it was also the first to evaluate the implications for patients with darker Fitz-Patrick skin types (III, IV, and V) using biometric assessment. Nevertheless, the small population of study subjects and the lack of a control group might limit any clinically meaningful conclusion from the data, yet as a pilot study, the results showed that the tested HA ECM provided a beneficial, durable treatment for the rejuvenation and augmentation of the labia majora with a good safety profile. These results should be confirmed with complementary studies using a larger sample size.

## Conclusions

Extracellular matrix-based hyaluronic acid products are potentially an efficient solution for female external genitalia rejuvenation and are affordable and minimally invasive. Based on the findings of this study, the main effect of the therapy is mostly the restoration of genital elasticity by promoting elastogenesis and collagenases in the injected areas. Visual improvement was another outcome that led to patient satisfaction. The main limitation of this study was its small sample size, which can affect the statistical reliability, and larger studies are needed to follow the preliminary findings of this pilot study.
